# Leadership competencies for dentists: What do undergraduate students think?

**DOI:** 10.1371/journal.pone.0310459

**Published:** 2024-09-17

**Authors:** Saira Atif, Ayesha Fahim, Asma Rafi Chaudhry, Daniyal Naeem Dar, Sofia Ali Syed, Sadia Rana

**Affiliations:** 1 Combined Military Hospital Lahore Medical College & Institute of Dentistry, Lahore, Pakistan; 2 National University of Medical Sciences, Rawalpindi, Pakistan; 3 University College of Dentistry, The University of Lahore, Lahore, Pakistan; 4 Islamic International Dental College, Riphah International University, Islamabad, Pakistan; 5 Dr Ishrat-ul-Ebad Khan Institute of Oral Health Sciences, Dow University of Health Sciences, Karachi, Pakistan; 6 Sharif Medical and Dental College, Lahore, Pakistan; University of the Witwatersrand Johannesburg Faculty of Health Sciences, SOUTH AFRICA

## Abstract

Dentists face difficult situations and challenges every day, but undergraduate dental students in Pakistan are not formally taught leadership skills or assessed for these skills. This study aims to explore the perception of undergraduate dental students on various leadership competencies. A multi-institutional cross-sectional observational study was conducted on undergraduate students in five institutions by using universal sampling technique. Students were asked to self-assess various leadership competencies for dentists based on 15 competencies using a validated questionnaire utilizing Likert scale response format. Responses to these items were presented as frequency and percentage. The overall scores were presented in mean and standard deviation and median and interquartile range. The differences in median scores of all items of the questionnaire between male and female students were determined with Mann Whitney U tests. The year-wise differences in the median of all response items of the questionnaire were determined using Kruskal Wallis H test. A total of 750 students, of which 570 (76%) were females and 180 (24%) were males, participated in the study. Most of the students (n = 708, 94.4%) did not attended any leadership course or workshop in the last five years. Majority of the students perceived that their leadership competencies were fair to excellent. Empathy was perceived as ‘excellent’ by the majority of the students (n = 294, 39.2%). Majority of the students (n = 319, 42.5%) perceived that their ‘authenticity’ skills were ‘good’. Statistically significant differences were reported between male and female students in most of the leadership skills including ‘compassion’, ‘advocacy skills’, ‘inquiry skills’, ‘empathy’, ‘integrity’, ‘ability to build trust with others’, ‘managing conflict’, ‘leading groups/teams’, ‘dealing with difficult personalities’, and ‘likelihood to exercise leadership during a crisis’ (p < 0.05). There were also significant differences in the median item scores in 13 out of 15 leadership domains and the year of study (p < 0.05). The study identified the perceptions of students regarding different leadership competencies. It gives indications that which leadership competencies need to be incorporated, promoted, and enhanced in leadership curriculum to make them effective dental practitioners and leaders in future. Incorporating these targeted leadership courses into the curriculum can provide participants with the opportunity to refine their existing leadership strengths and develop a well-rounded set of competencies essential for making a significant contribution in their chosen fields.

## Introduction

Leadership is the art of social influence, which motivates people and maximizes their efforts to achieve a goal [1]. The concept of leadership is defined and explained in various ways depending on the specific discipline and personal experiences. Leadership has received immense importance in healthcare services in recent years, as it has a major influence on patient safety, the quality of clinical care and the shaping of healthcare culture. The dental profession represents an integral component of healthcare services and is evolving at an incredible pace. There are various challenges dentists face as community leaders, health professionals, and educators. During events such as the recent Covid-19 pandemic, the need for effective leadership has increased as people face new challenges across healthcare professions and around the globe [2].

Effective leadership development is already an integral component of healthcare education worldwide [3]. Leadership development is a continuous learning process and involves a variety of forms of growth that promote and assist in leadership potential and abilities, including formal/informal and structured/unstructured activities [4]. These activities are directed at helping individuals being trained to translate newly learned skills to real and immediate situations. Various ethical dilemmas, team and system dynamics, cultural differences, and communication with patients have to be taught and practiced preparing students for leadership roles [5].

Training programs enhancing leadership qualities have been successful in college students. In healthcare professions, including dentistry, inadequate leadership negatively affects the proper functioning of a healthcare system [6]. Ineffective communication between dentists and stakeholders and a lack of community-oriented, socially aware, and culturally sensitive dentists are a few of the challenges being faced by the general public and healthcare professionals [7]. From a fresh graduates’ perspective, the ability to adequately manage a dental practice after graduation and having expertise in financial matters are integral to the success of dentists. Practicing dentists also consider staff management as the most difficult part of running a dental practice [8]. Practice management and leadership courses improve the perceptions of students about their skills. Studies have highlighted the importance of leadership skill development programs catering to a diverse group of undergraduate dental students.

Finding the equilibrium between clinical care, practice management, and leadership roles is an increasing concern. A curriculum incorporating leadership courses can cater to a diverse group of students in order to address their immediate learning needs and expand their horizons [9]. Leadership is considered an integral part of curricula at all levels of education [10]. Local programs can cater to a diverse group of students with or without prior leadership experience and training. These programs should address students’ immediate learning needs and include topics that expand their horizons.

The Bachelor of Dental Surgery (BDS) course is a four-year degree program taught at undergraduate dental colleges in Pakistan. The curriculum is based on the guidelines provided by the Pakistan Medical Commission. Leadership development has received little attention in Pakistan [11]. There is a lack of well-structured learning and training opportunities for the development of leadership and practice management skills in undergraduate dental students in Pakistan. Leadership is not offered as a separate module in undergraduate dental courses. It is a part of the behavioral sciences course which caters to leadership development in the areas of ethical practices, conflict management, team building and communication skills through lectures, small group discussions and case scenarios. Behavioral science is taught during preclinical years 1 and 2 and assessed at the end of the second year with a written examination. During the clinical years (years 3 and 4), students start practicing their leadership skills mostly through direct communication and clinical encounters with patients. Their leadership and management skills are not formally taught or assessed in the clinical years. However, it is important for dental undergraduate students to develop these skills during their educational years. Reforms are being made to incorporate leadership in dental education curriculum in Pakistan for that, it is important to understand the perception of the students regarding their leadership needs.

In our local context, there are limited published studies on perceptions of leadership among undergraduate dental students. The perceptions of a group of students are affected by the environment, exposure, experiences and behaviors of all stakeholders. For a specific module/course on leadership and management to be designed, perceptions of dental students regarding leadership and its various aspects need to be understood. The current study aims to explore the perception of undergraduate dental students regarding various leadership competencies using a questionnaire.

## Materials and methods

### Study design and participants

This multi-institutional cross-sectional observational study was conducted from September 1 to December 15, 2022. Students from all four years of the BDS program, studying in five dental schools in Pakistan, were invited to participate in the study. Students who migrated from international institutes were excluded. Detained students (repeaters) were included because these students do not attend regular classes for the repeated year and only appear in end of the year professional university examinations for which universities only give two additional chances in the repeated year for clearing exam as per Pakistan Medical and Dental Council guidelines, after which the student is debarred from continuing dental education. Data were collected from dental students at the University of Lahore, Azra Naheed Dental College, Sharif Medical and Dental College, CMH Lahore Medica College & Institute of Dentistry National University of Medical Sciences, and Khyber Medical University. These institutions/universities were selected by non-probability convenience sampling method because of ease of accessibility and permission granted for data collection. These institutions house students from across the country; thus, the study participants were expected to represent the population of Pakistan. The total number of enrolled students was 900. All the enrolled students were considered our study sample using universal (census) sampling technique to justify the minimum sample required and for comprehensive data collection. The minimum sample size calculated using Cochran’s formula based on a previous study was 720 participants [12].

### Ethics statement

The entire study was conducted in accordance with the Declaration of Helsinki, and ethical approval was obtained from the Ethical Review Committee of the parent institution (600/ERC/CMH/LMC). The perceptions of leadership of undergraduate dental students were recorded by sending an online questionnaire developed on Google Docs via official email IDs along with consent form. The study design and its purpose were explained, contact numbers of the principal investigators were provided to ask any questions related to study. Rights of participation and withdrawal mechanism were provided. The students were briefed that the data were collected for research purpose only and that the results would not affect their grades in any way. The collected data were kept anonymous using fictitious numbers and the names of the institution were not asked in the questionnaire. Moreover, students were given the opportunity to withdraw at their will at any stage of the study.

### Questionnaire

The questionnaire consisted of four parts. Part one asked about demographic information, including sex and year of study, and inquired about prior experience of attending any leadership course or workshop (other than behavioral science courses) in the last five years. Part two was a 15-item questionnaire regarding a self-assessment of leadership competencies originally defined by Kalenderian et al. [8] The questionnaire utilized a 5-point Likert scale response format (very poor = 1, poor = 2, fair/moderate = 3, good = 4, excellent = 5). The maximum possible score for each skill was 5, and the minimum possible score was 1. Part three asked, ‘How likely do you think it will be that you continue to practice developing your leadership skills?’ The options were ‘very likely’, ‘somewhat likely’ and ‘unlikely’. The questionnaire was validated by two medical educationists and was piloted on five dental students prior to the study.

### Data analyses

The data were analyzed using SPSS version 27 (IBM, Chicago, IL, USA). Qualitative variables such as sex (male/female), year of study (1/2/3/4), and responses to individual items were expressed as frequencies and percentages. Responses to leadership-related questions using Likert format were expressed as median and interquartile range (IQR) and for ease of comparison also in mean and standard deviation (SD). Differences in medians between all the response items of the questionnaire and sex were determined with Mann Whitney U tests. Year-wise differences in the median of all response items of the questionnaire were determined using Kruskal Wallis H tests. In all analyses, a p < 0.05 was taken as statistically significant.

## Results

Of the 900 students who were given the questionnaires, 750 completed them (response rate = 83.3%). The reliability was high (Cronbach’s alpha 0.903). Of the 750 BDS students, 570 (76%) were females and 180 (24%) were males. There were 195 (26%) students from year 1, 180 (24%) from year 2, 189 (25.2%) from year 3, and 186 (24.8%) from year 4. The majority of the students (n = 708, 94.4%) did not attend any leadership course or workshop in the last five years.

Students’ perceptions of their leadership skills are given in Table 1. Compassion, empathy, ability to influence others, self-management, relationship management, authenticity, ability to build trust with others, personal responsibility, and leading groups/teams were ranked ‘good’ by the majority of the students. Integrity was perceived as ‘good’ or ‘excellent’ by equal proportions of participants. Majority of students perceived their advocacy, inquiry, conflict management, dealing with difficult personalities, and likelihood to exercise leadership during a crisis skills as ‘fair’.

**Table 1:** Frequency of responses of dental students about their leadership skill perception.

Statement	Very poor n (%)	Poor n (%)	Fair n (%)	Good n (%)	Excellent n (%)
Compassion	18 (2.4)	39 (5.2)	234 (31.2)	315 (42)	144 (19.2)
Advocacy skills	8 (1.1)	94 (12.5)	330 (44)	234 (31.2)	84 (11.2)
Inquiry skills	10 (1.3)	71 (9.5)	348 (46.4)	225 (30)	96 (12.8)
Empathy	-	21 (2.8)	138 (18.4)	297 (39.6)	294 (39.2)
Ability to influence	17 (2.3)	88 (11.7)	255 (34)	291 (38.8)	99 (13.2)
Self-management	18 (2.4)	96 (12.8)	204 (27.2)	294 (39.2)	138 (18.4)
Relationship management	35 (4.7)	58 (7.7)	231 (30.8)	294 (39.2)	132 (17.6)
Authenticity (transparency)	6 (0.8)	12 (1.6)	165 (22)	319 (42.5)	248 (33.1)
Integrity	5 (0.7)	19 (2.5)	150 (20)	288 (38.4)	288 (38.4)
Ability to build trust with others	11 (1.5)	52 (6.9)	177 (23.6)	275 (36.7)	235 (31.3)
Personal responsibility	15 (2)	39 (5.2)	174 (23.2)	291 (38.8)	231 (30.8)
Managing conflict	11 (1.5)	67 (8.9)	267 (35.6)	249 (33.2)	156 (20.8)
Leading groups/teams	29 (3.9)	94 (12.5)	231 (30.8)	276 (36.8)	120 (16)
Dealing with difficult personalities	20 (2.7)	76 (10.1)	303 (40.4)	237 (31.6)	114 (15.2)
Likelihood to exercise leadership during a crisis	21 (2.8)	81 (10.8)	264 (35.2)	255 (34)	129 (17.2)

The median scores in majority of the leadership skills were perceived as ‘good’. Overall mean scores of students were higher for ‘empathy’ (4.15 ± 0.82) and the lowest for ‘advocacy’ (3.39 ± 0.88) as given in Table 2.

**Table 2:** Students’ perceptions of their current overall leadership skills as assessed by the questionnaire.

Leadership skill	Median score (IQR)	Mean (SD)
Compassion	4 (3–4)	3.70 (0.92)
Advocacy skills	3 (3–4)	3.39 (0.88)
Inquiry skills	3 (3–4)	3.43 (0.88)
Empathy	4 (4–5)	4.15 (0.82)
Ability to influence	4 (3–4)	3.49 (0.94)
Self-management	4 (3–4)	3.58 (1.01)
Relationship management	4 (3–4)	3.57 (1.02)
Authenticity (transparency)	4 (4–5)	4.05 (0.83)
Integrity	4 (4–5)	4.11 (0.86)
Ability to build trust with others	4 (3–5)	3.89 (0.97)
Personal responsibility	4 (3–5)	3.91 (0.96)
Managing conflict	4 (3–4)	3.63 (0.96)
Leading groups/teams	4 (3–4)	3.49 (1.03)
Dealing with difficult personalities	3 (3–4)	3.47 (0.96)
Likelihood to exercise leadership during a crisis	4 (3–4)	3.52 (0.99)

A comparison of the perceptions of leadership skills between female and male students shows that the perceived leadership skills scores of male students were significantly different than those of females in ‘compassion’, ‘advocacy skills’, ‘inquiry skills’, ‘empathy’, ‘integrity’, ‘ability to build trust with others’, ‘managing conflict’, ‘leading groups/teams’, ‘dealing with difficult personalities’, and ‘likelihood to exercise leadership during a crisis’ as indicated in Table 3. Majority of male and female students perceived their advocacy skills were ‘fair’. In case of ‘leading groups/teams’, majority of the male and female students perceived their skills as ‘good’. Those who perceived this skill as ‘excellent’ in ‘leading group/team’ domain, 45 (25%) male students perceived their skills were ‘excellent’ compared to only 75 (13.2%) of the female students. Similar trend was seen for ‘likelihood to exercise leadership during a crisis’.

**Table 3:** Frequency of responses of dental students about their leadership skills and comparison of median item scores based on sex of the participants.

Statement	Sex	Very poor n (%)	Poor n (%)	Fair n (%)	Good n (%)	Excellent n (%)	p value
**Compassion**	M	1 (0.6)	8 (4.4)	60 (33.3)	60 (33.3)	51 (28.3)	**0.047** *
	F	17 (3)	31 (5.4)	174 (30.5)	255 (44.7)	93 (16.3)	
**Advocacy skills**	M	-	9 (5)	78 (43.4)	69 (38.3)	24 (13.3)	**<0.001** *
	F	8 (1.4)	85 (14.9)	252 (44.2)	165 (28.9)	60 (10.5)	
**Inquiry skills**	M	-	9 (5)	81 (45)	63 (35)	27 (15)	**0.005** *
	F	10 (1.8)	62 (10.9)	267 (46.8)	162 (28.4)	69 (12.1)	
**Empathy**	M	-	6 (3.3)	39 (21.7)	85 (47.2)	50 (27.8)	**0.001** *
	F	-	15 (2.6)	99 (17.4)	212 (37.2)	244 (42.8)	
**Ability to influence**	M	8 (4.4)	16 (8.9)	54 (30)	66 (36.7)	36 (20)	0.051
	F	9 (1.6)	72 (12.6)	201 (35.3)	225 (39.5)	63 (11.1)	
**Self-management**	M	6 (3.3)	18 (10)	57 (31.7)	71 (39.4)	28 (15.6)	0.441
	F	12 (2.1)	78 (13.7)	147 (25.8)	223 (39.1)	110 (19.3)	
**Relationship management**	M	9 (5)	21 (11.7)	54 (30)	60 (33.3)	36 (20)	0.441
	F	26 (4.6)	37 (6.5)	177 (31.1)	234 (41.1)	96 (16.8)	
**Authenticity (transparency)**	M	-	-	42 (23.3)	72 (40)	66 (36.7)	0.256
	F	6 (1.1)	12 (2.1)	123 (21.6)	247 (43.3)	182 (31.9)	
**Integrity**	M	-	6 (3.3)	30 (16.7)	60 (33.3)	84 (46.7)	**0.020** *
	F	5 (0.9)	13 (2.3)	120 (21.1)	228 (40)	204 (35.8)	
**Ability to build trust with others**	M	-	6 (3.3)	39 (21.7)	70 (38.9)	65 (36.1)	**0.011** *
	F	11 (1.9)	46 (8.1)	138 (24.2)	205 (36)	170 (29.8)	
**Personal responsibility**	M	6 (3.3)	6 (3.3)	51 (28.3)	71 (39.4)	46 (25.6)	0.070
	F	9 (1.6)	33 (5.8)	123 (21.6)	219 (38.4)	186 (32.6)	
**Managing conflict**	M	1 (0.6)	11 (6.1)	66 (36.7)	47 (26.1)	55 (30.6)	**0.013** *
	F	10 (1.8)	56 (9.8)	201 (35.3)	202 (35.4)	101 (17.7)	
**Leading groups/teams**	M	3 (1.7)	15 (8.3)	48 (26.7)	69 (38.3)	45 (25)	**<0.001** *
	F	26 (4.6)	79 (13.9)	183 (32.1)	207 (36.3)	75 (13.2)	
**Dealing with difficult personalities**	M	3 (1.7)	18 (10)	60 (33.3)	63 (35)	36 (20)	**0.013** *
	F	17 (3)	58 (10.2)	243 (42.6)	174 (30.5)	78 (13.7)	
**Likelihood to exercise leadership during a crisis**	M	8 (4.4)	10 (5.6)	45 (25)	72 (40)	45 (25)	**<0.001** *
	F	13 (2.3)	71 (12.5)	219 (38.4)	183 (32.1)	84 (14.7)	

M: male; F: female; Mann Whitney U test used for comparison of median scores;

*p value significant at 0.05.

Table 4 presents year-wise responses to the leadership skill items. For year-wise comparison, the majority of the year 1 students felt that their ‘compassion’ skills were ‘good’ compared to year 4 students who majority of them perceived as ‘fair’. Majority of students from year 1 to year 4 perceived their ‘advocacy’ and ‘inquiry’ skills as ‘fair’. Majority of year 1 and year 4 students perceived their ‘empathy’ skills as excellent compared to students from year 2 and year 3. Majority of year 1 students perceived their ‘authenticity (transparency)’, ‘integrity’, ‘ability to build trust with others’, and ‘personal responsibilities’ skills were ‘excellent’ compared to students from other years. Kruskal Wallis H tests were conducted to explore the year-wise differences in the median leadership skills scores. The perceptions of year 1 to year 4 students were significantly different for the majority of leadership skills, as shown in Table 4.

**Table 4:** Frequency of responses of dental students about their leadership skills and comparison of median item scores based on year of study of the participants.

Leadership skill	Year of study	Very poor n (%)	Poor n (%)	Fair n (%)	Good n (%)	Excellent n (%)	p value
**Compassion**	1	-	6 (3.1)	42 (21.5)	102 (52.3)	45 (23.1)	**<0.001** *
	2	10 (5.6)	14 (7.8)	72 (40)	69 (38.3)	15 (8.3)	
	3	5 (2.6)	13 (6.9)	57 (30.2)	87 (46)	27 (14.3)	
	4	3 (1.6)	6 (3.2)	63 (33.9)	57 (30.6)	57 (30.6)	
**Advocacy skills**	1	-	18 (9.2)	87 (44.6)	72 (36.9)	18 (9.2)	**<0.001** *
	2	8 (4.4)	31 (17.2)	84 (46.7)	45 (25)	12 (6.7)	
	3	-	21 (11.1)	78 (41.3)	66 (34.9)	24 (12.7)	
	4	-	24 (12.9)	81 (43.5)	51 (27.4)	30 (16.1)	
**Inquiry skills**	1	-	12 (6.2)	84 (43.1)	69 (35.4)	30 (15.4)	**0.001** *
	2	6 (3.3)	24 (13.3)	84 (46.7)	48 (26.7)	18 (10)	
	3	1 (0.5)	17 (9)	84 (44.4)	57 (30.2)	30 (15.9)	
	4	3 (1.6)	18 (9.7)	96 (51.6)	51 (27.4)	18 (9.7)	
**Empathy**	1	-	6 (3.1)	27 (13.8)	69 (35.4)	93 (47.7)	**<0.001** *
	2	-	6 (3.3)	45 (25)	75 (41.7)	54 (30)	
	3	-	9 (4.8)	42 (22.2)	77 (40.7)	61 (32.3)	
	4	-	-	24 (12.9)	76 (40.9)	86 (46.2)	
**Ability to influence**	1	-	18 (9.2)	51 (26.2)	87 (44.6)	39 (20)	**<0.001** *
	2	3 (1.7)	24 (13.3)	81 (45)	63 (35)	9 (5)	
	3	5 (2.6)	19 (10.1)	66 (34.9)	78 (41.3)	21 (11.1)	
	4	9 (4.8)	27 (14.5)	57 (30.6)	63 (33.9)	30 (16.1)	
**Self-management**	1	-	12 (6.2)	36 (18.5)	105 (53.8)	42 (21.5)	**<0.001** *
	2	6 (3.3)	30 (16.7)	60 (33.3)	54 (30)	30 (16.7)	
	3	3 (1.6)	27 (14.3)	57 (30.2)	64 (33.9)	38 (20.1)	
	4	9 (4.8)	27 (14.5)	51 (27.4)	71 (38.2)	28 (15.1)	
**Relationship management**	1	6 (3.1)	9 (4.6)	45 (23.1)	111 (56.9)	24 (12.3)	**0.045** *
	2	5 (2.8)	22 (12.2)	69 (38.3)	54 (30)	30 (16.7)	
	3	6 (3.2)	18 (9.5)	63 (33.3)	63 (33.3)	39 (20.6)	
	4	18 (9.7)	9 (4.8)	54 (29)	66 (35.5)	39 (21)	
**Authenticity (transparency)**	1	3 (1.5)	-	27 (13.8)	87 (44.6)	78 (40)	**<0.001** *
	2	3 (1.7)	6 (3.3)	69 (38.3)	63 (35)	39 (21.7)	
	3	-	6 (3.2)	33 (17.5)	84 (44.4)	66 (34.9)	
	4	-	-	36 (19.4)	85 (45.7)	65 (34.9)	
**Integrity**	1	3 (1.5)	3 (1.5)	12 (6.2)	81 (41.5)	96 (49.2)	**<0.001** *
	2	2 (1.1)	10 (5.6)	66 (36.7)	57 (31.7)	45 (25)	
	3	-	6 (3.2)	39 (20.6)	66 (34.9)	78 (41.3)	
	4	-	-	33 (17.7)	84 (45.2)	69 (37.1)	
**Ability to build trust with others**	1	3 (1.5)	6 (3.1)	27 (13.8)	78 (40)	81 (41.5)	**<0.001** *
	2	4 (2.2)	17 (9.4)	57 (31.7)	66 (36.7)	36 (20)	
	3	1 (0.5)	11 (5.8)	69 (36.5)	53 (28)	55 (29.1)	
	4	3 (1.6)	18 (9.7)	24 (12.9)	78 (41.9)	63 (33.9)	
**Personal responsibility**	1	3 (1.5)	3 (1.5)	27 (13.8)	78 (40)	84 (43.1)	**<0.001** *
	2	3 (1.7)	18 (10)	33 (18.3)	88 (48.9)	38 (21.1)	
	3	3 (1.6)	6 (3.2)	60 (31.7)	53 (28)	67 (35.4)	
	4	6 (3.2)	12 (6.5)	54 (29)	72 (38.7)	42 (22.6)	
**Managing conflict**	1	-	24 (12.3)	57 (29.2)	66 (33.8)	48 (24.6)	0.434
	2	4 (2.2)	14 (7.8)	75 (41.7)	52 (28.9)	35 (19.4)	
	3	1 (0.5)	20 (10.6)	60 (31.7)	71 (37.6)	37 (19.6)	
	4	6 (3.2)	9 (4.8)	75 (40.3)	60 (32.3)	36 (19.4)	
**Leading groups/teams**	1	-	27 (13.8)	60 (30.8)	78 (40)	30 (15.4)	**0.003** *
	2	4 (2.2)	29 (16.1)	57 (31.7)	66 (36.7)	24 (13.3)	
	3	7 (3.7)	14 (7.4)	51 (27)	81 (42.9)	36 (19)	
	4	18 (9.7)	24 (12.9)	63 (33.9)	51 (27.4)	30 (16.1)	
**Dealing with difficult personalities**	1	3 (1.5)	15 (7.7)	75 (38.5)	72 (36.9)	30 (15.4)	0.315
	2	3 (1.7)	24 (13.3)	72 (40)	57 (31.7)	24 (13.3)	
	3	5 (2.6)	13 (6.9)	90 (47.6)	60 (31.7)	21 (11.1)	
	4	9 (4.8)	24 (12.9)	66 (35.5)	48 (25.8)	39 (21)	
**Likelihood to exercise leadership during a crisis**	1	3 (1.5)	12 (6.2)	69 (35.4)	72 (36.9)	39 (20)	**0.002** *
	2	2 (1.1)	31 (17.2)	72 (40)	51 (28.3)	24 (13.3)	
	3	7 (3.7)	17 (9)	75 (39.7)	66 (34.9)	24 (12.7)	
	4	9 (4.8)	21 (11.3)	48 (25.8)	66 (35.5)	42 (22.6)	

Kruskal Wallis H test used for comparison of median scores;

*p value significant at 0.05.

Students were asked the question, “How likely do you think you will continue to practice developing your leadership skills?” Of the 750 respondents, the majority of the students (n = 405, 54%) believed that there was a likelihood of continuous development of leadership skills, 318 (42.4%) participants thought that there was ‘somewhat’ a likelihood of continuous development of their leadership skills, whereas only 27 participants (3.6%) believed that it was unlikely for them to practice developing their leadership skills.

## Discussion

Leadership is an important skill for a graduating dentist, but it is not being formally taught to students in this part of the world. Dentists face difficult situations and challenges every day, and it has been recommended that dental students be trained accordingly. This study was conducted to increase awareness and attract the attention of the masses to the understanding of undergraduate dental students’ perceptions of leadership qualities.

In this study, a vast majority of the participants did not attend any courses on leadership off campus. This was considered to remove the confounding variable and to explain the context of study participants. A voluntary leadership development program was offered in 2008 at the Case School of Dental Medicine, which proved to be beneficial for future dentists [13]. The Harvard School of Dental Medicine included a formal course on leadership theory and skills in 2010 [8]. The developed world soon integrated leadership skills into its dental curricula and is now formally assessing those skills as well [14]. The developing world, however, did not pay much attention to formally teaching and assessing these soft skills until recently. In the last couple of years, literature has emerged on the need to analyze leadership qualities among dental students. A study in India concluded that dental students do not have sufficient management and leadership skills [15]. Only recently did a study in Pakistan explore the essential non-cognitive skills necessary for a dental graduate, which included leadership and management skills, among others [16]. Given the extreme dearth of knowledge about the topic in the developing world, the results of this study will pave the way for future studies incorporating leadership in dental students.

In this study, the best-ranked leadership quality was empathy, followed by integrity. Empathy is an essential component of healthcare practice, without which any practicing clinician cannot become a successful professional. The largest number of students felt they had excellent empathy, which points towards the fact that the aspiring dentists were already aware of their professional responsibilities. A study conducted in 2019 showed that the empathy level of undergraduate dental students in Pakistan significantly declined from their preclinical years to their clinical years [17]. A study conducted in Saudi Arabia claimed that the empathy level increased from the first year until the final year of dentistry [18]. A study conducted on Polish students concurred that the empathy level of final-year dental students decreased in comparison to students in earlier years [19]. There are several other studies which presented a general decrease in empathy [20–22]. However, it is interesting to note that all of the year 4 students in this study perceived their empathy as ‘fair’ to ‘excellent’. Moreover, majority of the year 1 and year 4 students felt that their empathy skills were ‘excellent’ whereas majority of year 2 and year 3 students perceived their empathy skills as ‘good’. This decline could be attributed to the increase in workload or technical skill-centered curriculum during these years where they are transitioning into skill development during which students could be overburdened by their clinical rotations along with the arduous curriculum; as a result, they pay little attention to empathic patient care [20]. More attention needs to be given to the leadership skills of undergraduate students in order to produce empathic healthcare providers.

Several studies have been conducted in the past to highlight the importance of leadership and other related soft skills in graduating dentists. In fact, the General Dental Council of Britain has included ‘management and leadership’ as one of their four main achievable competencies for dental graduates [2]. After the inclusion of this competency, all European countries strived to achieve it in their own way. This brings to light the dilemma in underdeveloped and some developing countries where such competencies are not considered mandatory by governing bodies [23]. In Tunisia, the clinical skills of graduating dental students were rated far better than their management and leadership skills [24]. Similar results were reported in studies from India, East Africa, Malaysia and Nepal, among other countries [25–28]. However, in this study, in all leadership domains, majority of the students perceived their skills from fair to excellent which represents that they are well aware of the importance of these skills and the taught dental curriculum has given them enough informal exposure.

Considering the scope of leadership in clinical and academic dental practice, the findings of this study show that most students are aware of the various attributes of leadership. Students not only recognize its importance but also know that formal training for leadership is required during their study period. However, do the students also practice these leadership skills when dealing with the patients or in their everyday learning, need to be assessed as well. It is time to bring major reforms to undergraduate dental programs related to leadership skill assessment and involve the government and all stakeholders in forming policies for future healthcare providers.

In this study, most of the students were interested in leadership skill development in future which corroborates with previous studies [29]. It can be concluded that suitable courses can be added to their syllabi to enhance their existing attributes and to develop sound professionals who can contribute positively to the field. This also highlights the role of academia in training students to develop evidence-based leadership skills. Such studies will help educators design a culturally and contextually appropriate leadership curriculum for dental students.

Comparison between perception of male and female students have highlighted some interesting results. Male students’ perception of ‘excellent’ skills in ‘leading groups/teams’, ‘managing conflict’, ‘dealing with difficult personalities’, and ‘likelihood of exercising leadership during a crisis’ in comparison to females could be due to their past experiences of witnessing less females in national workforce, more opportunities available to exercise these leadership skills in our cultural context to male students, and also because majority of the leadership roles in private and government dental healthcare sectors are held by men compared to women [30, 31]. Moreover, females who perceived that their ‘empathy’ skills were ‘excellent’ were more in comparison to males. Similar results were also reported by others [17, 18, 32].

This study had a few limitations. We did not collect qualitative data from students, which could explain their perceptions of leadership skills. Future studies should include responses from all stakeholders, including faculty members, upper management and even patients. Additionally, quasi-experimental studies could be conducted to acquire perceptions of students of leadership courses. The results of this study should be dealt with caution as the perceptions of undergraduate students in a subset of Pakistani population may not be generalized. The aim of this study was to record the perceptions of the students and these perceptions could be influenced by personal biases or self-presentation. Perceptions are based on individual interpretations and experiences which are not direct observations of their leadership competencies but did give a valuable input about the areas which needed attention in the curriculum and can help tailor leadership assessments in future to focus on specific competencies or address misconceptions.

## Conclusions

Most undergraduate dental students in Pakistan have not studied leadership as part of a formal course. According to their self-assessments, students scored highest in ‘empathy’ and lowest in ‘advocacy’. Even with good scores in self-assessment, the majority of the students expressed that they are likely to develop their leadership skills in the future. The results indicate that despite not having a formal module on leadership, students perceive their leadership competencies from fair to excellent in all these leadership competencies. Formal training and assessment may further help to evaluate and improve their leadership skills. The study identified the perceptions of students regarding different leadership competencies. It gives indications that which leadership competencies need to be incorporated, promoted, and enhanced in leadership curriculum to make them effective dental practitioners and leaders in future. Incorporating these targeted leadership courses into the planned curriculum can provide participants with the opportunity to refine their existing leadership strengths and develop a well-rounded set of competencies essential for making a significant contribution in their chosen field.

## Supporting information

S1 Dataset Study participants original data set. (XLSX)
